# Effects of a Trans-Theoretical Model-Based Health Education Program on the Management of Cognitive Dysfunction in Older Adults With Mild Cognitive Impairment: Study Rationale and Protocol Design for a Randomized Controlled Trial

**DOI:** 10.3389/fpsyt.2020.616420

**Published:** 2021-01-13

**Authors:** Xiaoshen Liu, Lina Wang, Hong Tao, Chenxi Ge, Xueting Zhen, Xue Sun, Simeng Wang, Liming Su

**Affiliations:** ^1^School of Medicine, Huzhou University, Huzhou, China; ^2^School of Medicine, Huzhou University, Huzhou Central Hospital, Huzhou, China; ^3^AdventHealth Whole-Person Research, Orlando, FL, United States

**Keywords:** cognitive dysfunction, self-management, trans-theoretical model, health education, mild cognitive impairment

## Abstract

**Introduction:** Recent studies have confirmed that the management of cognitive dysfunction produces considerable positive effects in individuals with mild cognitive impairment (MCI), however, compliance with participation in various cognitive dysfunction management strategies remains scant in older adults with MCI. Health education programs can improve the level of knowledge of the disease effectively, though it remains unclear as to whether health education programs are sufficient to promote behavior changes of older adults with MCI in the community.

**Objective:** The study aims to provide insight into the effect of a trans-theoretical Model (TTM)-based health education program on increasing knowledge about mild cognitive impairment (MCI), compliance in cognitive dysfunction management, and other cognition-related health outcomes (general cognitive function, sleep quality, depression symptoms, apathy symptoms) for older adults with MCI.

**Methods:** This study is a single-blinded, randomized, prospective clinical trial. We will recruit 132 participants with MCI who will be randomly assigned to a TTM-based health education group and a standard health education group in a ratio of 1:1. The intervention group will receive a TTM-based health education program (1 session/week, 4–560 min/session for 8 weeks), while the control group will receive standard health education. Assessors blinded to participant allocation will conduct baseline, post-intervention, and 3-month follow-up assessments. Statistical analyses will consist of the Wilcoxon test, the Chi-square test, the *T*-test, and 2 (group) × 3 (time) ANOVA with a 5% cut-off for significance.

**Discussion:** Supposing the TTM-based health education program will provide validated community-based cognitive dysfunction management strategies for older adults with MCI, this would be a feasible approach to improve the compliance of participation in cognitive dysfunction management and the cognition-related health outcomes.

**Clinical Trial Registration:** ChiCTR1900028351. Registered on December 19, 2019. http://www.chictr.org.cn/edit.aspx?pid=47223&htm=4.

## Background

Dementia, which remains incurable using existing technologies, has become a global health crisis. Recently, clinical research and basic research have focused on the preclinical stage of dementia—mild cognitive impairment (MCI) (1). MCI denotes problems with memory, attention, or other cognition, which are greater than expected for healthy aging but insufficient to interfere with daily activities (2). The clinical outcomes of MCI may remain stable, progress to dementia, or return to neurological integrity. The annual conversion rates from MCI to dementia are estimated from 10 to 59.4%, whereas 14.4 to 55.6% of those with MCI revert to normal cognition (NC) (3). The higher rates of MCI reversion emphasize the importance of early prevention and treatment at the MCI stage to forestall further cognitive decline.

Drug treatments resulted in no effect in any of the MCI studies (*N* = 53,693 patients, 95% CI: 0.000 to 0.005) (4), meaning that recent efforts have focused on non-pharmacological cognitive dysfunction management strategies, which have been proved promising for delaying cognitive decline, including physical exercise, cognitive training, musical therapy, mental/social activity, eating habits, and multi-domain interventions (5, 6).

In fact, older adults with MCI have poor compliance with participation in these cognitive dysfunction management strategies, with an average non-adherence rate of ~25% (7). There are many factors that influence compliance, including patients' lack of understanding of their disease, lack of social support, low sense of self-efficacy, and financial constraints (8). A promising intervention to improve compliance and educational strategies would help participants understand health information, and this may be essential to help individuals generate the motivations, beliefs and improve their overall compliance behavior (7, 9).

Health education can improve individual's level of disease knowledge, and it is an essential component of chronic disease management strategies. Health education helps individuals to understand the essence of disease management and encourages individuals to participate in their own disease management (10, 11). Nevertheless, previous studies have shown that older adults in the community still lack knowledge about the cause, diagnosis, and treatment of cognitive impairment. A total of 55.5% of community-dwelling older adults did not perceive themselves as very knowledgeable about dementia; more than 65% thought dementia was caused by healthy aging (12, 13). Meanwhile, older adults with MCI had lower scores with knowledge of dementia than those with normal cognition, and the scores would decrease as cognitive impairment worsened gradually (14). In addition, some limitations also occurred in health education activities aimed at the population with cognitive dysfunction, educators placing more emphasis on patients with dementia and their caregiver rather than those with MCI; the lack of health education programs for older adults with MCI in the community setting (15); and the failure to assess the extent of behavioral change caused by a health education (16). Anghel et al. found that better compliance behaviors were related to increased knowledge of the disease (17). Nevertheless, whether health education can improve the compliance of participation in cognitive dysfunction management for older adults with MCI is not clear.

The trans-theoretical model (TTM) is an integrative model of behavior change that has been applied successfully to improve compliance, self-management, and health behavior in older adults and patients with chronic diseases (18–20). The effectiveness of behavioral changes may increase, framing them with the TTM (18). TTM emphasizes behavior change as a process rather than a single event, which posits that individuals move through five stages of adoption health-promoting behaviors (i.e., pre-contemplation, contemplation, preparation, action, and maintenance) (21). Nevertheless, no education program based on TTM has been proven to promote behavior change and other cognition-related health outcomes on older adults with MCI.

To address this gap, we developed a TTM-based health education program, which was targeted to improve the disease knowledge and the compliance of participation in cognitive dysfunction management for older adults with MCI.

Our study seeks to answer two primary questions:

(1) What are the impacts of a TTM-based health education program on older adults with MCI? We hypothesize that this program would positively affect the level of compliance of participation in cognitive dysfunction management, disease knowledge, and other cognition-related health outcomes (general cognitive function, sleep quality, depression symptoms, apathy symptoms) for older adults with MCI compared to the control group.

(2) Is it feasible to use the TTM-based health education program for recruitment and compliance in the community setting? We hypothesize that older adults with MCI would be unlikely to refuse to participate in this program and would have high compliance, and no adverse events would occur during the intervention.

## Materials and Methods

### Study Design and Participants

This study is a randomized controlled trial (RCT, Clinical Trials.gov number: ChiCTR1900 028351) with a 3-month follow up. This protocol has been approved by an independent ethics committee (Third People's Hospital of Huzhou, Zhejiang Province). It will be conducted in accordance with the ethical principles of the Declaration of Helsinki. The participants will be recruited from the community's health care center in Huzhou, Zhejiang Province, China. This program will consist of two arms: the intervention group (TTM-based health education program) and the control group (standard health education).

Our staff will distribute recruitment leaflets at community health care centers and local senior centers. Local health care providers will also help to make referrals to our study. Individuals who show interest will be invited for an in-person interview to screen for eligibility by three trained staff and a trained neurologist-psychiatrist will examine the potential participants to provide the final diagnosis of MCI. In the first round of eligibility assessments, potential participants complaining of memory impairment will be recruited. The second round of eligibility assessments will include demographic data, sensory function evaluation, cognitive function, and functional evaluation of activities of daily and medical history.

Participants will be eligible for the trial if they are 60 years of age or older with Petersen criteria for MCI (22). Participants who have severe physical disorders or mental disorders at present, or who have unfortunate events during the trial or follow-up period will not continue to receive the health education program and will be withdrawn from the study. Detailed inclusion and exclusion criteria are listed in Table 1. All participants will be fully informed of the program protocol, and only those who sign the informed consent before baseline will be included in this study. They will receive a gift for the completion of each session of the TTM-based health education program.

**Table 1:** Inclusion criteria and exclusion criteria.

**Inclusion criteria**	**Exclusion criteria**
■ Aged 60 years or over ■ Meet diagnostic criteria of MCI proposed by Petersen (22) • Chief complaint of memory impairment confirmed by self-report/relatives; • Objective cognitive impairment inconsistent with age and education Montreal Cognitive Assessment-Basic (MoCA-B) scores 18 for those with elementary education level, scores 21 for those with secondary education level, scores 23 for those with tertiary education level (23); • Preserved global cognitive function as evidenced by Mini-mental State Examination (MMSE) score ≥24 (24), and absence of dementia [diagnosis by a psychiatrist according to DSM-V (25)]; • Intact Activities of Daily Living (ADL) [ADL score was ≤ 16 (26)] ■ Absence of self-reported visual or auditory impairmen ■ Capacity to provide written informed consent ■ Willingness to participate in the entirety of the study	■ History of severe disease (e.g., severe cardiovascular and cerebrovascular diseases, cancer and other severe physical disease, psychiatric disorders (neurodevelopmental disorders, schizophrenia spectrum, major depression disorder, obsessive-compulsive and related disorders and other major psychotic disorders), terminal illness) ■ Serious events occurring during the intervention period (e.g., acute stroke, fall) ■ Lost to follow-up ■ Participation in another training related to cognitive function within 1 year

### Sample Size

Based on our pilot study, Cohen's *d* = 0.684, effect size =0.324. Cohen's d and the effect sizes were calculated for between-group differences of means of the level compliance of participation in cognitive dysfunction management using an independent-groups pre-test and post-test design (IGPP). Sample size estimation was conducted using statistical software G^*^power. The analysis, with an independent *t*-test using β = 0.95 and α = 0.05, requires a total sample size of 114 participants. Considering a 15% dropout rate, our total sample size comes to 132 participants (66 cases for both the intervention and control groups).

### Randomization, Allocation Concealment, and Blinding

After completing the screening, eligible participants will be allocated randomly to one of two groups: TTM-based health education program group (intervention group) or standard health education group (control group) in a ratio of 1:1 using a computer-generated random number list. The random allocation sequence will be sealed in opaque envelopes by a staff member who will be not involved in study intervention, screening assessments, data collection, or data analysis. Participants will be informed that they will learn facts and management strategies about MCI, and that they will be blinded as to whether they receive health education based on TTM or standard health education. The data collectors and the data analyst will be blinded to group allocations.

### Intervention

#### Intervention Group

**Figure 1:**
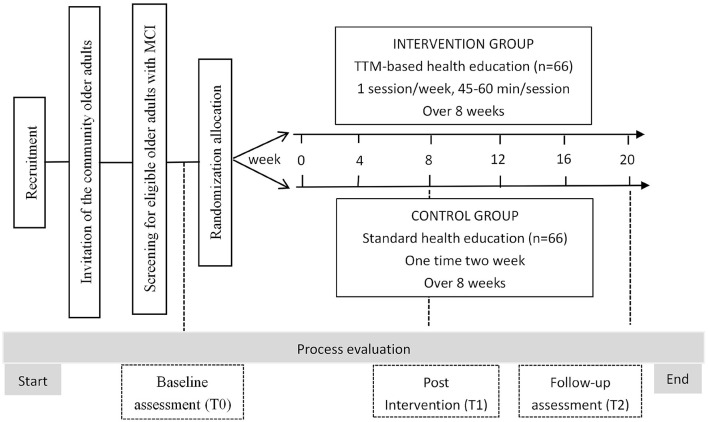
Overview of the study procedure.

The intervention group participants will attend one TTM-based health education session/week, 45–60 min/session for 8 weeks. The TTM encourages an assessment of an individual's current stage of behavior change and provides ten strategies to facilitate the construction of health education content according to the specific behavior stage (27). Therefore, before the intervention, the behavior stage of the participants' cognitive dysfunction management will be assessed by the researcher according to some questions (21) which will guide the researcher to match the appropriate health education contents (see Figure 1). After completing each health education session that matches the current behavioral characteristics, the participant's behavior stage of cognitive dysfunction management will be reassessed, and health education content will be adjusted accordingly. With any behavior change, relapses are common (28), and we will still re-assess the behavior stage and provide stage-matched health education content for participants. The content of health education sessions will be derived from evidence-based knowledge and recommendations will be founded on mild cognitive impairment guidelines (3). The details of this program are displayed in Supplementary Table 1.

#### Control Group

The control group participants will attend one health promotion session/2 weeks, 45 min/session for 8 weeks that will be delivered by the researcher in another community health care center. The health promotion session will also be derived from evidence-based knowledge and recommendations (3). Each health education session covers different subjects, including basic knowledge of MCI, e.g., the prevalence of MCI, risk factors of MCI, symptoms of MCI, consequences and impacts of MCI, development, and diagnosis of MCI; management strategies of MCI, e.g., taking part in regular exercise (twice/week), encouraging participation in reading, puzzle games, and other cognitive activities, learning basic knowledge of a healthy diet and recommending a Mediterranean diet. For any questions asked by the control participants, general advice, without giving information relating to the behavior change stage and corresponding health education, will be given for ethical consideration.

**Figure 2:**
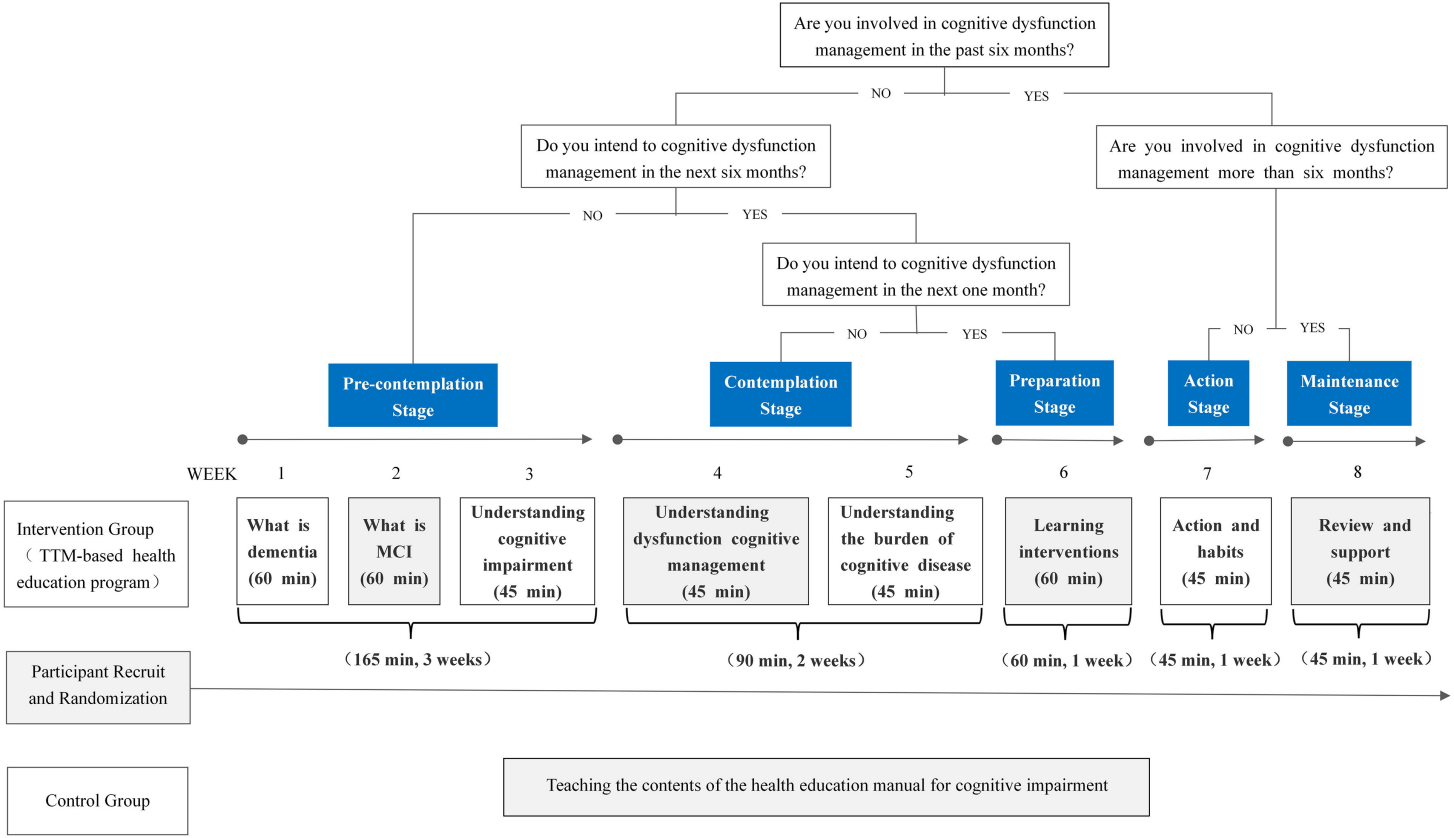
A flow chart of conducting the TTM-based health education program.

The staff will record attendance for each participant each session over the 8-week intervention period and ask about the presence of any adverse effects, such as pain or discomfort at each session. The interventions will be delivered at the different sites to minimize experimental contamination among the participants. The study procedure is shown in Figure 2.

### Outcomes and Measuring Instruments

#### Socio-Demographic Profile

A self-designed sheet will be used to collect the demographic data, including age, gender, education, job, financial status, marital status, living condition, family history of dementia, amount of smoking, amount of alcohol consumption, eating habits, physical activities, cognitive activities, physical health status, and clinical subtype of MCI [tested by the Auditory Verbal Learning Test (Chinese Version) (29) according to the Winblad criteria (30)].

### Primary Outcomes

#### Level of Compliance of Participation in Cognitive Dysfunction Management

The compliance of participation in cognitive dysfunction management will be determined using the Cognitive Dysfunction Management Compliance Scale (31). This scale was developed by our research team. It is composed of six dimensions: (1) perceived susceptibility (seven items); (2) perceived seriousness (four items); (3) perceived benefits (three items); (4) perceived barriers (seven items); (5) cues to action (three items); and (6) self-efficacy (eight items). Each item is scored based on a five-point Likert scale (1=not at all agree; 2=disagree; 3=not concerned; 4=agree; 5=very agree), (–) for reverse counting. A higher score means more adherence to cognitive management. The Cronbach's α coefficient of the overall questionnaire was 0.904, and content validity I-CVI was 0.92.

#### Level of Knowledge of MCl

The scores of MCI knowledge will be determined using the MCI Knowledge Questionnaire. The questionnaire, designed by HY, Pan of Fudan University (32), has three domains: (1) basic knowledge of MCI (four items); (2) risk factors of MCI (eight items); and (3) prevention, treatment, and cognition of MCI (eight items). The answer for each item is true (1) or false (0). Total scores on the questionnaire range from 0 to 20, with higher scores indicating more knowledge. The Cronbach's α coefficient of the overall questionnaire was 0.85, the content validity I-CVI was 0.93 (32).

### Secondary Outcomes

#### General Cognitive Function

The cognitive function will be assessed using the Chinese version of the Montreal Cognitive Assessment Basic (MoCA-B). It was developed as a collaborative project between Canada and Thailand to differentiate between older adults with and without MCI regardless of level of literacy. MoCA-B includes nine domains: executive functioning, memory, language, orientation, counting, abstract, visual perception, naming, and attention (33). According to the Chinese cultural background, the optimal cutoff scores for MCI screening were 19 for individuals with no more than 6 years of education, 22 for individuals with 7 to 12 years' education, and 24 for individuals with more than 12 years of education. The Cronbach's α was reported at 0.807, and the criterion-related validity was 0.787 (25).

#### Theory of Mind

Affective and cognitive Theory of mind (ToM) will be assessed using the “Yoni task” (Chinese version) (34). A total of 103 pictures can be subdivided into three types that correspond to affective ToM, cognitive ToM, and control conditions. A face named “Yoni” is shown in the middle with all pictures. The participant will be asked to complete special tasks depending on Yoni's gaze direction, facial expression and the information contained in the sentence. Correct answers received one point and incorrect answers received zero points (35).

#### Depression Symptoms

Depressive symptoms will be assessed using the Chinese version of the 15—item Geriatric Depression Scale (GDS). The total score ranges between 0 and 15, using seven as the cutoff point, with higher scores indicating severe depression. It has been validated in Chinese populations with reliability (Cronbach's α was 0.89) and validity was 0.96 (36).

#### Apathy Symptoms

Apathy symptoms will be assessed using the 18-item Apathy Evaluation Scale (AES), which involves cognitive, behavioral, emotional, and “other.” Scores range from 18 to 72 with higher scores corresponding to more severe apathy (37). It has been validated in the Chinese older adult population with Cronbach's α 0.89, test retest reliability 0.88, and inter-tater reliability 0.86 (38).

#### Sleep Quality

The Pittsburgh Sleep Quality Index (PSQI) has been widely used to measure subjective sleep quality in older adults with MCI (39, 40). It involves seven components: sleep quality, sleep latency, sleep duration, habitual sleep efficiency, sleep disturbances, use of sleep medication and daytime dysfunction (41). Each component is reflected by a score ranging from 0 to 3, total scores range from 0 to 21. Higher scores indicate worse subjective sleep quality. It has been validated in a Chinese population with Cronbach's α 0.82−0.83, and the test-retest 0.85 (42).

#### Data Collection

Before randomized allocation, the baseline data (T0) will be collected at a community health care center by the staff blinded to group allocation, including socio-demographic profile, primary outcomes and secondary outcomes; primary outcomes, and secondary outcomes will be measured again at the end of the intervention (T1) and 3 months after intervention (T2). Table 2 shows the assessment and timeline of the study.

**Table 2:** Summary of study assessments and time-lines.

	**Questionnaire items**	**Time point**				
		**Baseline**	**Randomized allocation**	**Intervention**	**Post intervention**	**Follow-up 3 months**
		**T0**			**T1**	**T2**
Enrollment						
Eligibility screening						
Informed consent						
Screening	**General information**					
	Demographic information	✓				
	**Daily functions**					
	Activities of Daily Living (ADL)					
	**Neuro psychological examination**					
	Mini-Mental State Examination (MMSE)					
	Montreal Cognitive Assessment Basic (MoCA-B)					
	Auditory Verbal Learning Test (AVLT)					
Measurements						
Primary outcomes	**Compliance of cognitive dysfunction management**					
	Cognitive Dysfunction Management Compliance Scale	✓			✓	✓
	**Level of knowledge of MCl**					
	MCI Knowledge Questionnaire	✓			✓	✓
Secondary outcomes	**General cognitive function**					
	Montreal Cognitive Assessment-Basic (MoCA-B)	✓			✓	✓
	Theory of Mind (ToM)	✓			✓	✓
	**Neuropsychiatric symptoms**					
	Depression Scale Short-form (GDS-15)	✓			✓	✓
	Apathy Evaluation Scale (AES)	✓			✓	✓
	**Sleep quality**					
	The Pittsburgh Sleep Quality Index (PSQI)	✓			✓	✓

#### Retention Strategies

Several safeguards will be put in place to decrease the dropout rate. (1) collecting multiple contact information, including contact information of a secondary contact person; (2) distributing a gift for time spent at baseline and follow up interviews; (3) enrolling in WeChat Group (WeChat, a mobile application, which is an instant messaging application) and receiving reminder messages of upcoming intervention; (4) making multiple contact attempts to complete each intervention; and (5) providing a quiet and comfortable environment for intervention.

### Statistical Analysis

Data collection will be conducted in a single room and kept in a password protected computer folder. Collected data will be encoded in Excel and double-checked. Data processing and statistical analysis will be conducted using SPSS 22.0. Descriptive statistics will summarize demographic data and observable variables using frequencies and percentages for categorical variables, means ± standard deviations for continuous variables. We will employ exploratory analysis (normality testing and distribution curves) to examine the distribution of data. The proportionality of baseline data for both groups will be compared using the non-parametric Wilcoxon test, the Chi–square test, and the *T*-test. A 2 (group) × 3 (time) ANOVA will be used to determine the effect of the group factors, the intervention group and control group differences within-group over time, and interaction effects. Simple effect analysis will be added when there is an interaction between group factors and time factors. The intention-to-treat principle will follow data analysis. A two-tail *P*-value of < 0.05 will be considered statistically significant.

## Discussion

Although older adults are at risk of dementia, older adults with MCI progress to dementia more quickly than do those who are cognitively intact (43). The symptoms of early MCI are not visible, and most individuals are hidden in the community (44). One primary challenge in implementing health education in the community is the lack of cognitive health education programs for older adults with MCI. Given limited community health care resources in China, only a few large cities can provide professional cognitive training and mental health therapists (45). It is essential, therefore, to design a cognitive health education program for older adults with MCI in the community.

Health education, as the non-pharmacological method, can improve health literacy; nevertheless, whether it can facilitate behavior change remains to be further investigated. This study innovatively uses the compliance of participation in cognitive dysfunction management as a primary indicator to assess of the effectiveness of health education interventions. This study will determine the effectiveness of a TTM-based cognitive health education program on increasing the compliance of participation in cognitive dysfunction management and disease knowledge for older adults with MCI.

TTM explains how behavior change occurs and offers explicit suggestions on how to help individuals change their behavior (21). In recent years, TTM also played an essential role in health education that promotes behavior change. Mary et al. (46) performed a randomized controlled trial of a TTM-based exercise stage-matched intervention with exercise behavior in sedentary older women. They found that, compared to the control group, the TTM-based intervention group demonstrated more willingness to start exercising, with longer adherence to an exercise, and higher exercise self-efficacy. Additionally, the authors claimed that health education based on TTM was considerably significant for future behavior changes. However, the effectiveness of a health education based on TTM for older adults with MCI is not well-understood.

Recent longitudinal studies identified depression and sleep complaints as remarkable risk factors to predict cognitive decline (39). Depressive symptoms, apathy symptoms and poor sleep are highly prevalent among older adults with MCI, with prevalence of 31.8, 44.8, and 63%, respectively (47–49). These conditions greatly interfere with the normal life of older adults with MCI. Some studies have shown that cognitive dysfunction management can significantly improve the neuropsychiatric symptoms (depression and apathy) and sleep quality (39). Considering the potential positive effect of TTM-based cognitive health education on cognitive dysfunction management behavior, further verification is needed to demonstrate whether health education can create favorable mental and physiological conditions to improve neuropsychiatric symptoms and poor sleep in older adults with MCI.

Retention and dropout are crucial issues for the intervention study. Since the TTM-based health education program will last for 8 weeks, we might have difficulty in recruiting participants and sustaining their attendance throughout the intervention period. The researcher will adopt retention strategies and work closely with community health care center staff.

There are some limitations to the current study. First, the sample population may not represent the overall characteristics of older adults with MCI, thereby limiting the generalizability of the TTM-based health education program. Second, self-reported subjective sleep quality will be measured in this study; therefore, the results may be influenced by the subjective factors of the participants. Third, the follow-up of health education in this study will be limited to 3 months. Hence, the more long-term effect of health education on cognitive dysfunction management compliance needs to be further explored. Future research is needed to explore the above research gaps. Finally, there are several limitations of TTM, which would not be avoided in the high level of social ecological effects. TTM ignores the social context in which change occurs, including socioeconomic status (SES) and income. In addition, this model assumes that individuals make coherent and logical plans in their decision-making process when this is not always true. In this study, the participants might be still in the initial stage of behavioral change after the end of the intervention period (8 weeks), however, we hope that the older adults will get some knowledge of MCI, and their self-efficacy to participate in the management of cognitive functions can be evoked by the TTM education program.

In summary, this study will provide insight into the effect of a TTM-based health education program on increasing knowledge about MCI and compliance in management of cognitive dysfunction. If proven effective, health care professionals and practitioners in the geriatric field will be provided with a validated community-based cognitive health education program and will be prompted to increase its application in older adults with MCI. The benefits of this program will be perceived over time with a trend toward increasing the knowledge of MCI, which may lead to higher compliance with cognitive dysfunction management and improvements in global cognitive function.

## Trial Status

The study is currently ongoing. Recruitment began in July 2020 and will conclude in May 2021.

## Ethics Statement

Medical Ethics Committee of the Third People's Hospital of Huzhou, Zhejiang Province has approved and regulates the ethical execution of this research (number 2019-080). The patients/participants provided their written informed consent to participate in this study.

## Author Contributions

LW: research concept, research design, and critical revision of the manuscript for intellectual content. XL: drafting the first version of the manuscript, submitting the manuscript for publication, and assisting in the development of the research design. HT: contributing to the design of the study. CG and XZ: assisting with statistical analytic planning. XS, SW, and LS: assisting with participant enrolment, consenting, and data acquisition plan. All authors contributed to the design and drafting of the manuscript, read, and approved the final manuscript.

## Conflict of Interest

The authors declare that the research was conducted in the absence of any commercial or financial relationships that could be construed as a potential conflict of interest.
