# Supplementary material for: Effects of a Trans-Theoretical Model-Based Health Education Program on the Management of Cognitive Dysfunction in Older Adults With Mild Cognitive Impairment: Study Rationale and Protocol Design for a Randomized Controlled Trial
Source: Front Psychiatry. 2021 Jan 13;11:616420. doi: 10.3389/fpsyt.2020.616420 (PMC7838522; doi:10.3389/fpsyt.2020.616420)
Supplement: Supplementary file 1 [file Data_Sheet_1.PDF]

frontiers

Supplementary Material

1 Supplementary Table

Supplement Table 1 Details of trans-theoretical model-based health education program

Behavior Stage	Goal	Health Education Theme	Intervention Strategies
**Pre-contemplation**	To increase awareness of the need to behavior change for conducting cognitive dysfunction management	1.What is dementia	① Use PowerPoint presentation to explain the types, symptoms, and treatments of dementia.<br>② Provide information about the impact of dementia on caregivers, family, society and economy.
		2.What is MCI	① Use PowerPoint presentation to explain the types, early symptoms, risk factors, protective factors of MCI.<br>② Ask participants to sit in a circle and talk about their unhealthy lifestyle related to MCI.
		3.Understanding cognitive impairment	① Use PowerPoint presentation to explain the relationship between aging, MCI, and dementia.<br>② Encourage participants to think about the impact of behavior change on cognitive function.
**Contemplation**	To increase motivation and confidence to change behavior for conducting cognitive dysfunction management	4.Understanding cognitive dysfunction management	① Use PowerPoint presentation to explain the cognitive benefits of adopting cognitive dysfunction management.<br>② Improve participants' confidence and self-efficacy to participate in these cognitive management well.<br>● *take some successful cases which improved cognition through cognitive dysfunction management.*<br>③ Discuss the reasons with participants for not conducting cognitive dysfunction management.
			① Use PowerPoint presentation to explain the burden on dementia care and the

		5.Understanding the burden of cognitive disease	costs of dementia treatment.<br>② State the facilitating factors on conducting cognitive dysfunction management.<br>● *understand its potential benefits for cognition.*<br>● *understand the adverse outcomes of disease development.*<br>③ Encourage participants to reflect on their cognition' level through assessing their cognitive function (e.g., executive function, delayed recall, attention), and improve the understanding about importance of cognitive dysfunction management.
**Preparation**	To negotiate a plan for conducting cognitive dysfunction management	6.Learning interventions	① Use PowerPoint presentation to explain how to conduct the exercise intervention, cognitive training and dietary methods that are beneficial to cognitive dysfunction.<br>② Encourage participants to take action after they have learned some cognitive dysfunction management methods.<br>③ Formulate some schemes with participants jointly for conducting cognitive dysfunction management.<br>④ Make a promise to peers that they will conduct cognitive dysfunction management, which will strengthen the belief about behavior change.
**Action**	To establish behavior habits of conducting cognitive dysfunction management	7.Action and habits	① Use visual and verbal cues to determine whether participants implement cognitive dysfunction management as planned.<br>● *ask participants about the methods and duration of physical activity, cognitive activity.*<br>● *ask peers or family members about the implementation of participants' cognitive dysfunction management.*<br>● *ask participants to submit daily logs related to cognitive dysfunction management.*<br>② Give the personalized feedback about the current cognitive dysfunction management behavior.<br>● *adjust the methods or intensity according to the conduction of the scheme.*<br>③ Encourage participants to speak about the problems that they encountered during conducting the cognitive dysfunction management and help them find some solutions, including<br>● *set an alarm clock for older adults who tend to forget to do exercise.*<br>● *organize groups for older adults who don't like to do exercise alone.*

**Maintenance**	To review the implementation of cognitive dysfunction management and keep the compliance	8.Review and support	① Compare changes in cognitive dysfunction after conducting cognitive dysfunction management; they will be aware of improvements in cognitive function and motivate them to keep it up.<br>② Integrate social support into the process of cognitive dysfunction management and assist participants in persisting in cognitive management.<br>● *community service center organize some group events, e.g., reading, calligraphy, festival events.*<br>● *community activity center provides some place and equipment for participants,e.g., table tennis table, dancing room, reading room.*<br>● *doctors works at community health care center' provide some physical examination for participants.*<br>③ Plan for resisting the temptation to skip cognitive dysfunction management.<br>● *call participants once a week to ask them about the implementation of cognitive dysfunction management.*<br>● *create some cognitive dysfunction management groups to improve peer motivation and compliance of cognitive dysfunction management.*

Note: Health education programme derived from the evidence-based knowledge and recommendations of practice guideline for mild cognitive impairment (MCI) developed by American Academy of Neurology (AAN)[1].

REFERENCE

1. Petersen RC, Lopez O, Armstrong MJ, Getchius TSD, Ganguli M, Gloss D, et al. Practice guideline update summary: Mild cognitive impairment: Report of the Guideline Development, Dissemination, and Implementation Subcommittee of the American Academy of Neurology. NEUROLOGY (2018) 90(3):126-135. doi:10.1212/WNL.0000000000004826
